# Clinical and Genetic Study of a Pseudo‐Dominant Primary Ciliary Dyskinesia Pedigree: The First DNAAF1‐Associated Family Reported in Chinese Population

**DOI:** 10.1002/mgg3.70264

**Published:** 2026-07-21

**Authors:** Zhuoyao Guo, Ping Li, Gaoli Jiang, Weicheng Chen

**Affiliations:** ^1^ Respirology Department Children's Hospital of Fudan University Shanghai China; ^2^ Cardiovascular Center Children's Hospital of Fudan University Shanghai China

**Keywords:** *DNAAF1* gene, inheritance pattern, primary ciliary dyskinesia, pseudo‐dominant inheritance

## Abstract

**Background:**

Primary ciliary dyskinesia (PCD) is a rare, genetically heterogeneous disorder typically inherited in an autosomal recessive pattern. Pseudo‐dominant inheritance is exceptionally uncommon and remains poorly characterized in PCD.

**Methods:**

We conducted a clinical and genetic study of a non‐consanguineous Chinese PCD pedigree with four affected individuals across two generations. Whole exome sequencing and Sanger sequencing were performed to identify pathogenic variants.

**Results:**

Two variants in *DNAAF1*, c.1930A>T (p.Arg644Ter) and c.1022_1023del (p.Gln341ArgfsTer10), were identified and shown to co‐segregate with the disease in a pseudo‐dominant pattern. The proband, her brother, and her two naturally conceived sons were all affected, with her parents and husband being unaffected carriers. Transmission electron microscopy revealed absence of both outer and inner dynein arms, and high‐speed video analysis demonstrated immotile or severely reduced ciliary beating. Intrafamilial phenotypic variability was notable, including distinct laterality defects (situs inversus, heterotaxy, and situs solitus) and varying degrees of pulmonary involvement.

**Conclusion:**

Our report provides the first description of pseudo‐dominant inheritance in PCD and expands the knowledge of the disease by offering detailed clinical and ciliary phenotyping.

## Graphical abstract

We report pseudo‐dominant PCD inheritance in a Chinese family due to novel *DNAAF1* mutations. Affected members across two generations showed significant variability in lung disease progression and visceral arrangement.

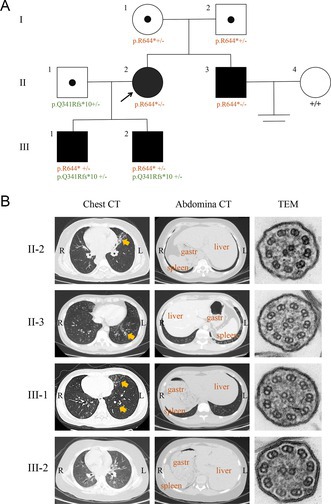

**Abbreviations**
CNKI: China National Knowledge Infrastructure
CT: Computerized tomography
HSVA: High‐Speed Video Analysis
IDA: Inner dynein arm
ODA: Outer dynein arm
PCD: Primary ciliary dyskinesia
TEM: Transmission electron microscopy
WES: Whole Exome Sequencing

## 1 Introduction

Primary ciliary dyskinesia (PCD) is a group of rare genetically heterogeneous disorders (~0.2/10,000–7/100,000 live birth), characterized by neonatal respiratory distress, recurrent respiratory tract infections, subfertility, and laterality defects (Shoemark et al. 2025; Ardura‐Garcia et al. 2020). To date, more than 50 ciliopathy‐associated genes have been identified as causative genes for PCD. Autosomal recessive inheritance is the predominant pattern in PCD, although autosomal dominant transmission occurs with *FOXJ1* mutations, and X‐linked inheritance is observed with *PIH1D3* and *OFD1* mutations (Despotes et al. 2024). Notably, pseudo‐dominant inheritance in PCD remains poorly characterized. In this study, we describe a Chinese PCD pedigree spanning two generations, with four affected individuals, and comprehensively analyze their clinical manifestations and genetic profile.

## 2 Methods

### 2.1 Subjects

The proband and her family members were enrolled at the Children's Hospital of Fudan University in October 2024. The study protocol was approved by the Ethics Committee of the Children's Hospital of Fudan University (Approval No. 2021–93). All participants, or their legal guardians for minors, provided written informed consent for the publication of case details and use of images.

### 2.2 Whole Exome Sequencing (WES) and Variant Analysis

Peripheral blood samples were collected from the proband and consenting family members. Genomic DNA was isolated using the Gene Blood DNA Rapid Extraction Kit (Qiagen, China). Whole‐exome capture and library preparation were performed using the SeqCap EZ MedExome kit (Roche NimbleGen) and KAPA Hyper Prep Kit (Roche KAPA), respectively. Sequencing was conducted on a HiSeq X Ten platform (Illumina) by Gemple Biotech Co. Ltd. (Shanghai, China). Raw reads underwent quality control and adapter trimming. The cleaned reads were aligned to the GRCh37/hg19 reference genome using BWA software (version 0.7.15). Variant calling and filtering were performed using established criteria as detailed in our earlier publication (Chen et al. 2024). Briefly, variants were excluded if they presented with a minor allele frequency ≥ 0.5% in public databases (gnomAD, ExAC, 1000 Genomes). Variants classified as benign or likely benign by multiple subscribers in the ClinVar database, synonymous variants, and intronic variants (located > 10 bp from splice sites) were also filtered out. Nonsynonymous missense variants predicted as benign or tolerated by all employed in silico tools, including SIFT, PolyPhen‐2, MutationTaster and CADD, were excluded. The remaining variants were further assessed according to the suspected inheritance pattern, previously reported pathogenicity, and the clinical phenotype of the affected individuals.

WES data were systematically interrogated for known PCD‐associated genes. The full list of PCD‐related genes included in the analysis is provided in Table S1. Candidate variants were classified according to the ACMG/AMP guidelines (Richards et al. 2015). Sanger sequencing was ultimately employed to confirm the candidate variants and examine co‐segregation within the family.

## 3 Results

### 3.1 Case Reports

**FIGURE 1:**
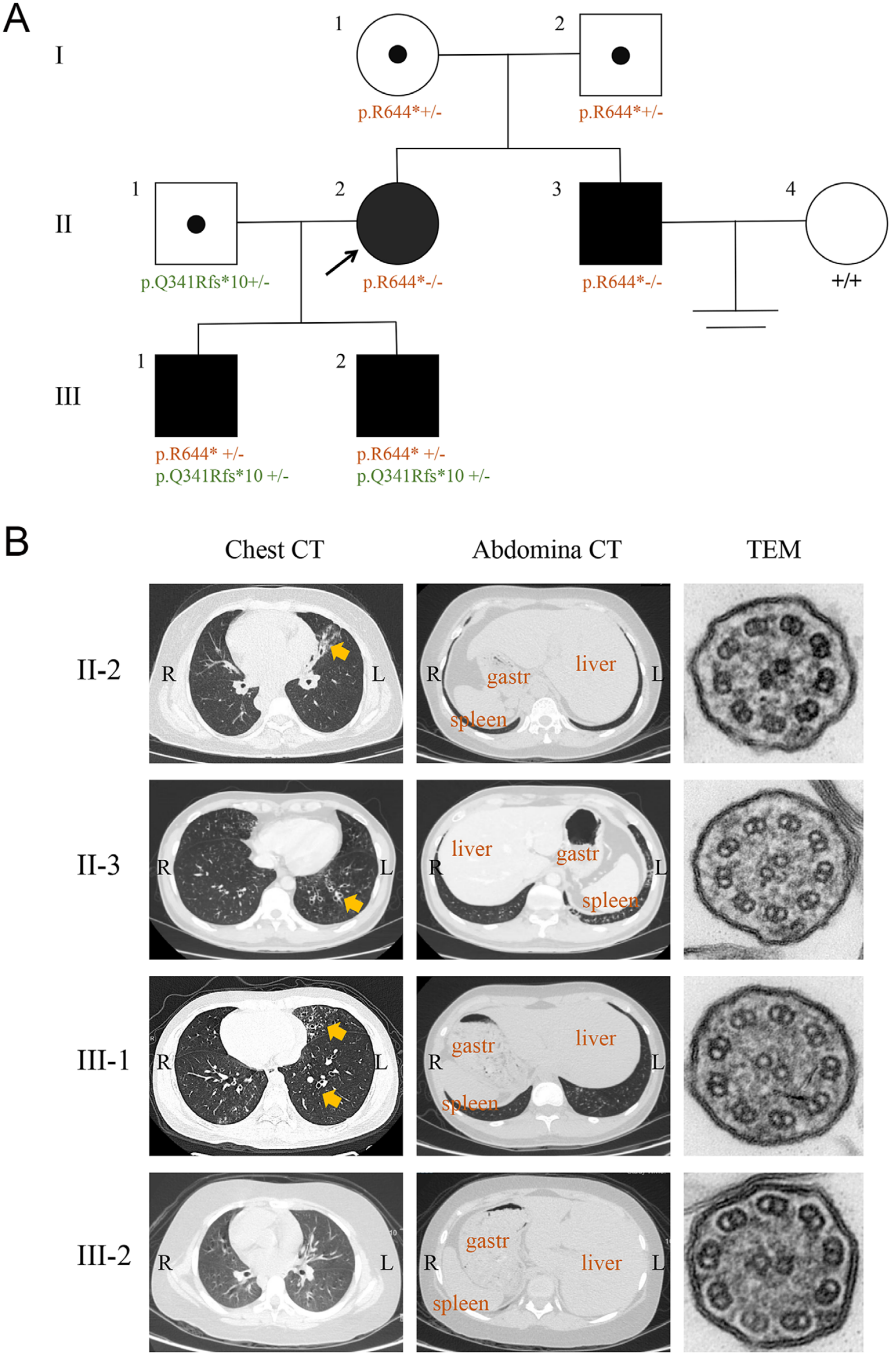
Genetic lineage and imaging features of the family. Pedigree structure of these family. (B) Representative images of thoracic CT, abdominal CT and TEM from II:2, II:3, III:1, and III:2. Chest imaging indicates the direction of the apex of the heart. The yellow arrow shows the location of the lung lesion. The position of the liver, stomach and spleen is shown in the abdominal CT. Abdominal imaging reveals inversus of liver and stomach, combine with right‐side polysplenia and dilated azygos vein for III:2. TEM image show absence of outer and inner dynein arm on the bottom. Scale bar, 50 nm.

During the treatment of a 40‐year‐old woman with chronic wet cough and heterotaxy in our center, PCD phenotypes were surprisingly identified in her brother and two sons. The family pedigree is presented in Figure 1A. The detailed clinical manifestations and latest medical examination of each patient are summarized in Table 1. Thoracic visceral inversus and inversus of liver and stomach, combine with right‐side polysplenia, absence of inferior vena cava and mild bronchiectasis of the middle lobe of the left lung were observed on thoracoabdominal imaging of the proband (II:2). About her younger brother (II:3), computerized tomography (CT) scan revealed bronchiectasis in left lower lobe of the lung and situs solitus. Chest imaging of proband's eldest son (III:1) showed bronchiectasis in multiple lobes of the lung. Intriguingly, further systemic evaluation revealed right isomeric broncho‐pulmonary morphology, combined with dextrocardia and abdominal visceral inversus. Mild bronchitis and situs inversus totalis were seen in her younger son (III:2) (Figure 1B).

**TABLE 1:** Clinical and genetic features of the patients.

Characteristics	II:2	II:3	III:1	III:2
	Homozygous mutations		Compound heterozygous mutations	
Gender	Female	Male	Male	Male
Age (years)	40	38	15	7
High (cm)	156	163	178	130
Weight (kg)	70	65	75	40
Birth status	Full‐term natural labor	Full‐term natural labor	34 weeks	Full‐term natural labor
Situs status	Heterotaxy	Situs solitus	Heterotaxy	Situs inversus totalis
Neonatal respiratory distress	No	No	Yes	No
Respiratory symptom	Yearround wet cough	Yearround wet cough	Yearround wet cough	Yearround wet cough
Number of acute pneumonias	3	More than 30	More than 20	4
Respiratory Microbiology	No	Pseudomonas aeruginosa	No	NA
Otitis media	Yes	Yes	No	Yes
Hearing problems	Yes	Yes	No	Yes
Sinusitis	Yes	Yes	Yes	Yes
Smell problems	Yes	No	No	Yes
FVC	78.2	91.9	86.7	83.9
FEV1, % pred	67.7	90.0	95.5	88.8
Chest imaging	Bronchiectasis	Bronchiectasis	Bronchiectasis	Bronchitis
Infertility	No	Yes	NA	NA
Nasal nitric oxide (nL/min)	6.9	16.5	6.3	4.3
TEM	ODA+IDA	ODA+IDA	ODA+IDA	ODA+IDA
HSVA	Minimal residual movements	Minimal residual movements	Immotile	Immotile
Allele 1	c.1930A>T,p.R644*	c.1930A>T,p.R644*	c.1930A>T,p.R644*	c.1930A>T,p.R644*
Allele 2	c.1930A>T,p.R644*	c.1930A>T,p.R644*	c.1022_1023del,p.Q341Rfs*10	c.1022_1023del,p.Q341Rfs*10

Abbreviations: FEV1, forced expiratory volume in 1 second; FVC, forced vital capacity; HSVA, high speed video analysis; IDA, inner dynein arm; NA, not available; ODA, outer dynein arm; TEM, transmission electron microscopy.

The nasal nitric oxide levels are presented in Table 1 and all are far below the recommended diagnostic cut‐off (77 nL/min) (Shoemark et al. 2025). Transmission electron microscopy (TEM) was performed and showed the loss of both outer and inner dynein arms (Figure 1B).

High‐speed video analysis showed that the nasal cilia were completely immotile in both III:1 (Video S1) and III:2 (Video S2). However, cilia from the II:2 (Video S3) and II:3 (Video S4) were in a bent position and showed minimal residual movements. A powerful beating stroke followed by a recovery stroke was observed in the proband's husband (II:1) (Video S5) and parents (I:1, I:2).

**FIGURE 2:**
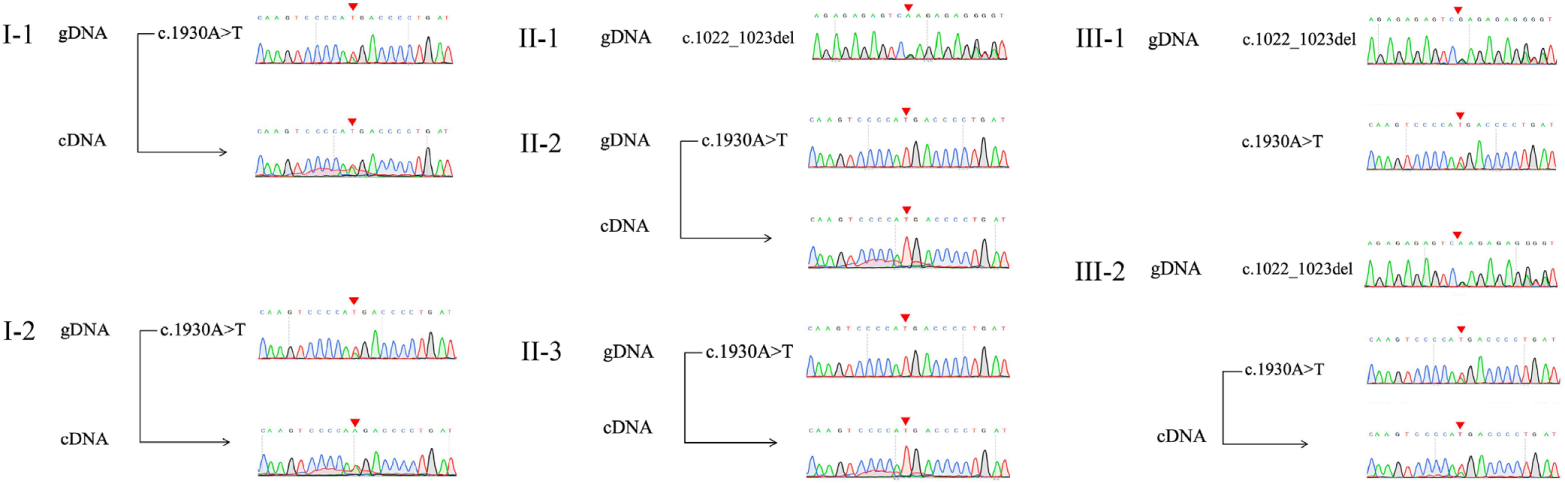
Sanger sequencing of the family.

The genetic analysis of the proband and her brother revealed two novel homozygous nonsense mutation (NM_178452.6: c.1930A>T, p.Arg644Ter) in *DNAAF1* gene. Compound heterozygous *DNAAF1* mutations c.1022_1023del/p.Gln341ArgfsTer10 and c.1930A>T/p.Arg644Ter were identified in her two sons. To further verify the variant and mode of inheritance, we performed Sanger sequencing in family members of proband (I:1, I:2, II:1 and II:3), and found that her husband was a carrier of c.1022_1023del/p.Gln341ArgfsTer10 intriguingly. Meanwhile, her parents were both found to be heterozygous for c.1930A>T, p.Arg644Ter, which supports an autosomal recessive inheritance pattern. The ACMG/AMP classification and supporting criteria for each identified variant are summarized in Table 2. The consequences of some of these mutations on the mRNA level were verified by sequencing of cDNA from nasal respiratory epithelial cells. c.1022_1023del was not detected at the mRNA level, which may result from nonsense‐mediated mRNA decay. The results of DNA and cDNA Sanger sequencing are presented in Figure 2. cDNA analysis could not be performed on II:1 and III:1 due to inadequate nasal epithelial cells.

**TABLE 2:** Summary of identified DNAAF1 variants, ACMG/AMP classification, and supporting evidence.

Position (GRCh37/hg19)	Transcript and exon	HGVS variant (c./p.)	ACMG/AMP classification	ACMG/AMP evidence criteria	PMID
Chr16:84199547‐84199548	NM_178452.6; exon7	c.1022_1023del; p.Q341Rfs*10	Pathogenic	PVS1 + PM2 + PM3 + PP4	27543293; 31772028
Chr16:84209770	NM_178452.6; exon11	c.1930A>T; p.R644*	Pathogenic	PVS1 + PM2 + PM3 + PP4	33942430

### 3.2 Literature Review

We searched PubMed, Web of Science, Springer, Embase, PubMed, Cochrane Library, Scopus databases, and CNKI for evidence relating to *DNAAF1* until December. 2025 using the search term “Kartagener syndrome”, “DNAAF1”, “LRRC50”, “primary ciliary dyskinesia” and “situs inversus”. After excluding articles with unclear or incomplete clinical information, 29 patients from nine countries including China, the United States, France, Japan, Korea, Pakistan, Spain, Belgium and Germany were identified (Zhou et al. 2020; Ito et al. 2024; Guo et al. 2020; Kim et al. 2023; Hartill et al. 2018; Loges et al. 2009; Duquesnoy et al. 2009; Raidt et al. 2014; Ahn 2020; Carretero‐Vilarroig et al. 2025; Zhou et al. 2025; Postema et al. 2020; Urbantat et al. 2023; Bolkier et al. 2022; Hao et al. 2021). These patients ranged from 3 to 58 years with an average age of 15.8 years. We then summarized the clinical features of patients with different variants in *DNAAF1* for comparison (Table 3). Overall, clinical and laboratory findings in PCD patients with *DNAAF1* mutations remain poorly documented in the literature. The majority of patients showed characteristic PCD manifestations, with situs inversus observed in 13 patients (54.2%) and heterotaxy in 4 patients (16.7%). TEM was done for 19 cases, in which 17 cases (89.5%) showed both ODA and IDA defects. HSVA was performed in 12 cases, all of which exhibited immotile cilia.

**TABLE 3:** Clinical data of all patients with *DNAAF1*.

Patient no	Age (years)	Ethnicity	Sex	Situs status	NRDS	Sinusitis	Otitis media	Wet cough	Infertility	FEV1, % pred	Bronchiectasis	CHD	nNO (nL/min)	TEM	HSVA	Allele 1	Allele 1
1 Zhou et al. (2020)	32	Chinese	Male	SIT	—	Yes	—	—	Yes	53.1%	Yes	—	—	ODA+IDA	—	c.509delG	c.[3G>A;124+1G>C]
2 Zhou et al. (2020)	37	Chinese	Female	SIT	—	Yes	—	—	Yes	23.8%	Yes	—	—	ODA+IDA	—	c.943A>T	c.943A>T
3 Ito et al. (2024)	58	Japanese	Female	SS	—	Yes	—	—	Yes	44.8%	Yes		20.7	ODA+IDA	—	c.86delG	c.86delG
4 Guo et al. (2020)	15	Chinese	Female	SIT	—	—	—	Yes	—	—	Yes	—	14.0	—	Immotile	c.376delG	c.1801_1804del
5 Kim et al. (2023)	4.5	Korean	Female	—	—	—	—	—	—	—	—	—	—	Normal		c.376del	c.376del
6 Kim et al. (2023)	3.8	Korean	Male	—	—	—	—	—	—	—	—	—	—	—	—	c.376del	c.1198_1199delinsG
7 Kim et al. (2023)	11.4	Korean	Male	—	—	—	—	—	—	—	—	—	—	—	—	c.1172C>T	c.1462C>T
8 Hartill et al. (2018)	—	Pakistani	Male	SS	—	—	—	—	—	—	—	—	10	ODA+IDA	Immotile	c.281delA	c.1484delC
9 Hartill et al. (2018)	—	Pakistani	Male	SA	—	—	—	—	—	—	—	Yes	—	ODA+IDA	—	c.281delA	c.1484delC
10 Hartill et al. (2018)	—	Pakistani	Male	SA	—	—	—	—	—	—	—	Yes	—	ODA+IDA	—	c.281delA	c.1484delC
11 Hartill et al. (2018)	—	Pakistani	Male	SIT	—	—	Yes	—	Yes	—	—	—	—	—	—	c.1484delC	c.1484delC
12 Hartill et al. (2018)	—	Pakistani	Male	SIT	Yes	Yes	—	—	—	—	—	Yes	—	ODA+IDA	Immotile	CNV	CNV
13 Hartill et al. (2018)	—	Pakistani	Female	—	—	—	—	Yes	—	—	—	No	—	ODA+IDA	Immotile	CNV	CNV
14 Loges et al. (2009)	—	American	Male	SIT	—	—	—	—	—	—	Yes	—	—	ODA+IDA	Immotile	c.811C>T	CNV
15 Loges et al. (2009)	4	German	Male	SS	—	—	—	—	—	—	Yes	—	—	ODA+IDA	Immotile	CNV	CNV
16 Loges et al. (2009)	5	Muslim	Female	SA	—	—	—	—	—	—	—	—	—	—	—	c.1698+1G> A	c.1698+1G> A
17 Duquesnoy et al. (2009)	—	French	Male	SS	—	Yes	—	Yes	—	—	Yes	—	—	ODA+IDA	Immotile	c.524T>G	c.524T>G
18 Duquesnoy et al. (2009)	—	French	Male	SS	—	Yes	—	Yes	—	—	Yes	—	—	ODA+IDA	Immotile	c.524T>G	c.524T>G
19 Duquesnoy et al. (2009)	—	French	Female	SIT	—	Yes	—	Yes	—	—	Yes	—	—	ODA+IDA	Immotile	c.115dupT	c.1300_1322del
20 Duquesnoy et al. (2009)	—	French	Female	SIT	—	Yes	—	Yes	Yes	—	Yes	—	—	ODA+IDA	Immotile	c.1198_1199insTCGC	c.124+1536_353‐2102del
21 Duquesnoy et al. (2009)	—	French	Female	SIT	—	Yes	—	Yes	Yes	—	Yes	—	—	ODA+IDA	Immotile	c.792C>A	c.508dupG
22 Raidt et al. (2014)	16	German	Female	SIT	—	—	—	—	—	—	—	—	—	ODA+IDA	—	c.1349_1350insC	c.1349_1350insC
23 Ahn (2020)	4	Asian	Male	SIT	Yes	Yes	—	Yes	—	—	—	—	—	—	—	c.376delG	c.1198_1199delinsG
24 Carretero‐Vilarroig et al. (2025)	7	Spanish	Male	SIT	Yes	Yes	No	Yes	—	—	No			ODA	Immotile	c.1300_1322del	c.1300_1322del
25 Zhou et al. (2025)	17	Chinese	Female	SS	—	Yes	Yes	Yes	—	—	Yes	—	11.0	—	—	c.376del	c.394del
26 Postema et al. (2020)	32	Belgian	Female	SIT	—	—	—	Yes	—	—	Yes	No	—	—	—	c.1528+2T>C	c.765C>G
27 Urbantat et al. (2023)	14	German	Female	SS	—	—	—	—	—	94.1%	—	—	6.0	ODA+IDA	—	c.1349dupC	c.1349dupC
28 Bolkier et al. (2022)	5	Israeli	Female	RAI	—	—	—	—	—	—	—	Yes	—	—	—	c.1698+1G>A	c.1698+1G>A
29 Hao et al. (2021)	3	Chinese	Male	—	—	—	—	—	—	—	—	—	—	—	—	c.1496del	c.1930A>T

*Note:* “—”, not reported.

Abbreviations: CHD, Congenital Heart Disease; CNV, Copy Number Variation; FEV1, Forced Expiratory Volume in 1 second; HSVA, High‐Speed Video Analysis; nNO, Nasal Nitric Oxide; NRDS, Neonatal Respiratory Distress Syndrome; SA, Situs Ambiguus; SIT, Situs Inversus Totalis; SS, Situs Solitus; TEM, Transmission Electron Microscopy.

## 4 Discussion

The airway epithelium is densely populated with motile cilia, each of which consists of nine peripheral microtubule doublets and two central microtubules. IDA and ODA project from peripheral doublets and produce ciliary beating waves via coordinated activation cycles (Legendre et al. 2021). A deficiency in ODA and/or IDA is the main cause of PCD. DNAAF1, a cytoplasmic protein essential for the preassembly of IDA and ODA, was first identified as a pathogenic gene for PCD in 2009 (Duquesnoy et al. 2009). In this study, we report a non‐consanguineous Chinese family with *DNAAF1* mutations.

As reviewed, c.1022_1023del was first described by Miao et al. (2016) in patients with neural tube defects without PCD phenotyping, and later by Blanchon et al. (2020) in a PCD patient with limited clinical details. c.1930A>T was reported by Hao et al. (2021) in a 0–3‐year‐old East Asian male with PCD. While the variants themselves are not novel, our study provides the detailed clinical and ciliary characterization that was previously lacking. First, we provide comprehensive pulmonary function data (FEV1, FVC), bronchiectasis status, nasal nitric oxide measurements, high‐speed video analysis of ciliary beating patterns, and transmission electron microscopy findings for all affected individuals across two generations. Notably, the 40‐year‐old female proband maintained nearly normal pulmonary function—a remarkable finding given that PCD typically demonstrates progressive annual decline in lung function (Davis et al. 2019). She reported infrequent pneumonia episodes, consistent with her chest CT showing only mild bronchiectasis with limited involvement. Second, we observed remarkable intrafamilial phenotypic variability in laterality. Within this single pedigree, the four affected individuals exhibited completely different visceral situs arrangements. This striking variability demonstrates that even within the same pedigree, the effects on left–right patterning can vary dramatically. Third, spontaneous conception in *DNAAF1*‐PCD has not been well documented in the literature. Having conceived two children without medical assistance, our proband (II:2) provides an example of preserved natural fertility in this genetic subtype, thereby expanding the reproductive phenotype spectrum of *DNAAF1* mutations and demonstrating that natural fertility is possible.

More importantly, this pedigree is particularly remarkable for its pseudo‐dominant inheritance pattern. This inheritance pattern has been rarely reported in diseases including Wilson's disease (Park et al. 2016), Gitelman's syndrome (de La Faille et al. 2011), and Friedreich's ataxia (Malaquias et al. 2023). It is well established that *DNAAF1* is transmitted as an autosomal recessive trait, and patients presenting a typical PCD phenotype are homozygous or compound heterozygous (i.e., bearing two pathogenic mutations in trans). In this “unlucky” family, the patient's husband happened to be a carrier of *DNAAF1* unexpectedly, resulting in the pseudo‐dominant inheritance of the autosomal recessive disease. Based on the estimated incidence of *DNAAF1*‐related PCD (approximately 1/444,663) and the population carrier frequency (approximately 1/334) (Hannah et al. 2022), the theoretical probability for a non‐consanguineous family to exhibit such a pseudo‐dominant inheritance pattern with affected offspring in the second generation is approximately 1 in 297 million (1/444,663 × 1/334 × 1/2), underscoring the exceptional rarity of this observation. To the best of our knowledge, it is the first time that pseudo‐dominant inheritance in PCD has been reported. This exceptionally rare inheritance pattern has direct and important implications for clinical genetic counseling. Under a typical autosomal recessive inheritance model, when one parent is affected by PCD and the other is an unrelated individual from the general population, offspring are generally expected to be asymptomatic carriers. However, in the context of pseudo‐dominant inheritance, if the healthy parent happens to carry a heterozygous pathogenic mutation in the same gene, the risk of having an affected child increases sharply to 50%. Our study is the first to demonstrate this pattern in the *DNAAF1* gene through complete family segregation analysis. Therefore, we recommend that in core families where one parent has a confirmed diagnosis of recessive PCD and has already given birth to affected children, carrier screening should be pursued in the healthy parent. Once pseudo‐dominant inheritance is confirmed, the recurrence risk should be reassessed.

In conclusion, we characterize the clinical and genetic features of a non‐consanguineous Chinese PCD family with *DNAAF1* mutations. Our study provides the detailed clinical and ciliary phenotyping that was previously lacking, including remarkable intrafamilial phenotypic variability in laterality and, for the first time, documented preserved natural fertility in a *DNAAF1*‐PCD female patient. Furthermore, this study provides the first report of pseudo‐dominant inheritance in PCD. These findings enrich the understanding of *DNAAF1*‐associated PCD and provide valuable evidence for future genetic counseling and personalized management of affected families.

## Author Contributions

All authors were involved in the critical revision of the manuscript. Z.G. and P.L. contributed to the study conception and design. G.J. and P.L. enrolled patients and collected clinical data. Z.G. analyzed clinical data and drafted the manuscript. W.C. had contributions to the revision of the manuscript, data re‐evaluation, and presentation. All authors approved the final version of the manuscript, including the authorship list.

## Funding

This work was supported by the National Natural Science Foundation of China (grant no. 82370309) and the Cross‐innovation Team Incubation Project of Children's Hospital of Fudan University (grant no. EKYX202402).

## Ethics Statement

The study protocol was approved by the Ethics Committee of the Children's Hospital of Fudan University.

## Consent

Written informed consent was obtained from all participants and the patients for publication.

## Conflicts of Interest

The authors declare no conflicts of interest.

## Supporting information


**Table S1:** PCD‐related genes included in WES analysis.

**Video S1:** High‐speed video analysis of nasal ciliary beating in individual III:1. The cilia are completely immotile.

**Video S2:** High‐speed video analysis of nasal ciliary beating in individual III:2. The cilia are completely immotile.

**Video S3:** High‐speed video analysis of nasal ciliary beating in individual II:2. Cilia are predominantly immotile, with only a few showing sluggish residual movement.

**Video S4:** High‐speed video analysis of nasal ciliary beating in individual II:3. Cilia are in a bent position and show minimal residual movement.

**Video S5:** High‐speed video analysis of nasal ciliary beating in the proband's husband (II:1). A powerful beating stroke followed by a recovery stroke is observed.

## Data Availability

The data that support the findings of this study are available on request from the corresponding author. The data are not publicly available due to privacy or ethical restrictions.
